# Eye Tracking Based Control System for Natural Human-Computer Interaction

**DOI:** 10.1155/2017/5739301

**Published:** 2017-12-18

**Authors:** Xuebai Zhang, Xiaolong Liu, Shyan-Ming Yuan, Shu-Fan Lin

**Affiliations:** ^1^College of Computer and Information Sciences, Fujian Agriculture and Forestry University, Fuzhou 350002, China; ^2^Department of Computer Science, National Chiao Tung University, Hsinchu 300, Taiwan

## Abstract

Eye movement can be regarded as a pivotal real-time input medium for human-computer communication, which is especially important for people with physical disability. In order to improve the reliability, mobility, and usability of eye tracking technique in user-computer dialogue, a novel eye control system with integrating both mouse and keyboard functions is proposed in this paper. The proposed system focuses on providing a simple and convenient interactive mode by only using user's eye. The usage flow of the proposed system is designed to perfectly follow human natural habits. Additionally, a magnifier module is proposed to allow the accurate operation. In the experiment, two interactive tasks with different difficulty (searching article and browsing multimedia web) were done to compare the proposed eye control tool with an existing system. The Technology Acceptance Model (TAM) measures are used to evaluate the perceived effectiveness of our system. It is demonstrated that the proposed system is very effective with regard to usability and interface design.

## 1. Introduction

Eye tracking technology, which is based on an eye tracker that measures the movement and positions of the eye has played an increasingly important role in psychology [1], marketing [2], and user interfaces [3]. Eye trackers have existed for a number of years, but, early in the development of the field of eye tracking, the use of eye trackers was largely confined to laboratory experiments to observe the nature of human eye movements, rather than to use these movements as an actual control medium within a human-computer interaction (HCI) [4]. Because the cost of eye trackers was around $30,000 a decade ago, it was too expensive to consider use in real user-computer interfaces. In recent years, with the development of better and cheaper components for gaze interaction, low-cost eye trackers [5] have been produced by several high-profile companies, such as Tobii's EyeX tracker [6], GazePoint's GP3 tracker [7], and the Eye Tribe Tracker [8]. As eye tracking gear gets cheaper, new applications with the concept of using eye tracking in HCI are clearly beginning to blossom [9–11].

Traditional user interfaces provide much more bandwidth from computer to user, such as images, animations, videos, and other media which can output large amounts of information rapidly. Whereas there are hardly any means of inputting comparably large amounts of information from users. The concept of HCI is to increase the bandwidth from user to computer with more natural and more convenient communication mechanisms. The eye is one of our mainly input mediums, and about 80 to 90 percent of the outside world information is obtained from the human eye [12]. For multimedia communication from user to computer, the eye movements can be regarded as a pivotal real-time input medium, which is especially important for people with motor disability (such as persons with Amyotrophic Lateral Sclerosis) [13]. The research of eye tracking technique in user-computer dialogue is mainly focused on incorporating eye movements into the multimedia communication with computer in a convenient and natural way.

Generally, the most intuitive solution for incorporating eye movements into user-computer dialogue would be substituted with an eye tracker directly for a manual input source, such as a mouse. By installing an eye tracker and using its *x*, *y* coordinate output stream as a virtual mouse, the moving of user's gaze would directly cause the mouse cursor to move. But the natural hand moving a mouse is very different from the eye movement to control virtual mouse. There are significant differences between the mouse and eye position to be considered in designing eye tracking based control system for user-computer dialogue. In order to provide appropriate communication, several improved eye tracking based control systems were developed. Nehete et al. [14] designed an eye tracking mouse that allows users to communicate with computer through active mobility such as eye movement or nose movement. Missimer and Betke [15] also constructed a system that uses the head position to control the mouse cursor and simulates the left-click and right-click of the mouse by blinking left or right monocular.

Although the eye control systems mentioned above are of benefit for users with physical or cognitive handicaps to interact with computers appropriately, the designed eye trackers are quite complicated and expensive. Users should wear inconvenient devices and make specific actions to control the system. To lower the threshold of usability for user, MastaLomaster [16] developed a prototype of eye control system that is based on low-cost eye trackers. The system supports most commercial low-cost eye trackers, such as Tobii EyeX and Eye Tribe. However, user should choose the desired function first and then do the real interaction with computer, which goes against user intuition and it is not natural to use.

In order to provide more natural and more convenient communication mechanisms, we present an eye tracking based control system for user-computer dialogue in this paper. The system combines both the mouse functions and keyboard functions, so that users can use our system to achieve almost all of the inputs to the computer without traditional input equipment. It was oriented towards improving the reliability, mobility, and usability for user to interact with computer by only using their eyes. The contributions of this work could be further described in detail by satisfying the following requirements:

1. Instead of using complicated designed equipment, the proposed system would provide extremely lightweight devices that are more convenient for user to use.
2. The proposed system is oriented towards the possibility of being used widely, which supports most of the low-cost eye trackers that are affordable for the majority of users.
3. The proposed system realizes all of the functions of regular input sources, including mouse and keyboard. User can efficiently interact with computer by only using their eyes.
4. The proposed system provides more natural and more convenient communication mechanism for user-computer dialogue and could also avoid annoying user with unwanted responses to their actions.

The remainder of the paper is organized as follows: in Section 2 related literature is reviewed in the area of eye tracking based communication system. The design and the detail workflow of the system functionalities will be described in Section 3. The experimental analysis and evaluations of the proposed system are presented in Section 4. Finally, Section 5 summarizes the paper contents and provides perspectives for future work.

## 2. Related Works

Generally, eye tracking measures the eyeball position and determines gaze direction of a person, and the movements of the eye can be tracked using different technologies. It can be categorized into four categories: infrared-oculography (IROG), scleral search coil method (SSC), electrooculography (EOG), and video-oculography (VOG). Currently, most of the eye tracking researches for HCI are based on VOG [17], because the VOG technique has minimized the invasiveness to user in some degree.

Chin et al. [18] proposed a cursor control system for computer users, which integrated the electromyogram signals from muscles in the face and point-of-gaze coordinates produced by an eye-gaze tracking system as inputs. Although it could facilitate a reliable click operation, it was slower than the control system that only used eye tracking and the accuracy was low. Missimer and Betke [15] constructed a system that uses the head position to control the mouse cursor and simulates left-click and right-click of the mouse by blinking left or right monocular. This system relied on the position of user's head to control the mouse cursor position. The irregular movement of user's head would affect the accuracy of click function. Lupu et al. [19] proposed a communication system for people with disabilities, which was based on a special designed device composed of a webcam mounted on glasses frame for image acquisition and processing. The eye movement is detected by the device and the voluntary eye blinking is correlated with a pictogram or keyword selection reflecting patient's needs. The drawback of this system is that the image processing algorithm could not accurately detect the acquired image with low quality and is not robust to light intensity. Later, to improve the reliability of the communication system they proposed an eye tracking mouse system using video glasses and a new robust eye tracking algorithm based on the adaptive binary segmentation threshold of the acquired images [20].

Lately, several similar systems were also developed by scholars [21, 22], and the main concept of these systems is to capture images from a camera either mounted on head gear worn by the user or mounted remotely and extract the information from different eye features to determine the point of the gaze. Since the commercial eye trackers were prohibitively expensive to use in HCI, all of the eye tracking control systems mentioned above were proposed with self-designed hardware and software. These systems were hard to achieve a widespread adoption, because the software and hardware design were closed source.

In recent years, several high-profile technology companies have developed commercial low-cost eye trackers, such as Tobii EyeX and Eye Tribe, which can be easily bought from the Internet. The low-cost eye trackers can be used as standard hardware for eye tracking control system. To lower the threshold of usability for user, MastaLomaster [16] developed a prototype of eye control system that is based on low-cost eye trackers and the released Application Program Interface (API). This system was composed of all of the functions of mouse and keyboard, and user should choose the desired function first and then do the real interaction with computer. The accuracy of the eye tracking control system will depend on the eye tracker employed. However, the usage flow of this system goes against user's intuition and it is not natural for user to communicate with computer smoothly. Therefore, in this paper we present an eye tracking based control system that not only supports the commercial low-cost eye trackers but also provides more natural and more convenient communication mechanisms for user-computer dialogue.

## 3. System Design

The proposed eye tracking based control system is a software application running on the low-cost eye trackers. The application detects users' gaze with “mouse cursor control” function of eye tracker. Mouse cursor control allows users to redirect mouse cursor to gaze position. Therefore, we will realize where user watches according to the position of mouse cursor. By gazing at the point for few seconds, the tool would generate the corresponding events. In this way, users can select and click the corresponding functions.

### 3.1. System Architecture

**Figure 1:**
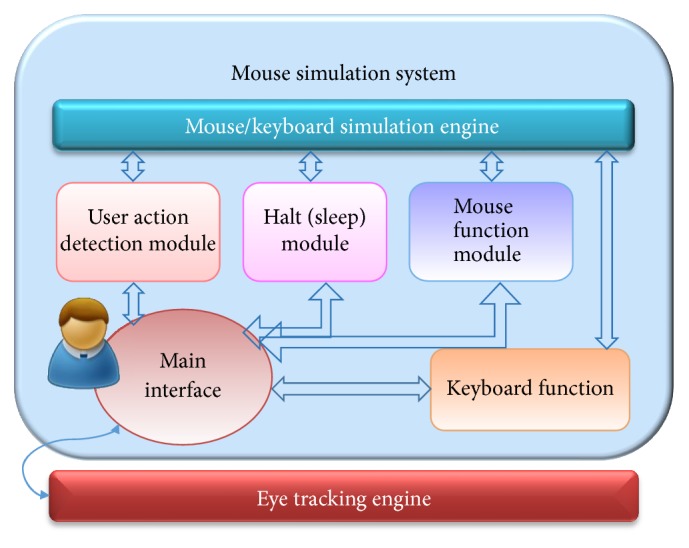
The architecture of the proposed system.

Figure 1 illustrates the system components and interactions of the proposed virtual mouse system. The software application is organized into two parts—the eye tracking engine part and mouse simulation part. The eye tracking engine part matches our software application with eye tracking device (such as Eye Tribe Tracker, which enables the eye to control the mouse cursor) to eye control mouse function. The mouse simulation part defines how the information provided by the mouse cursor is processed. This part consists of components including main interface, mouse/keyboard simulation engine, user action detection module, halt (sleep) module, mouse function module, and keyboard function module, as they are presented in Figure 1.

**Figure 2:**
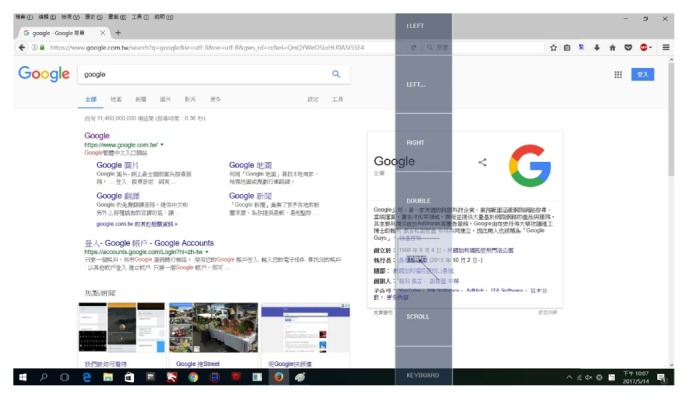
The screenshot of the toolbar for the proposed virtual mouse.

1. Main interface: it sets and manages the startup dialogue, provides user access to user action detection module, halt (sleep) module, mouse function module, and keyboard function module.
2. Mouse/keyboard simulation engine: it creates dynamic tool window, automatically changes the size of the tool window according to the screen resolution, and decides whether to display or obscure the toolbar; when the function menu is triggered, it sends the view point of user to the mouse function module; it receives information from modules and executes the corresponding mouse/keyboard events for users.
3. User action detection module: the fixation function in this module is defined to calculate the gaze time of the user and determines the mouse coordinates directed by eye movement. In addition, this module decides the function or key selected by the user and triggers the corresponding mouse/keyboard events.
4. Halt (sleep) module: it determines whether to stop eye tracking and enter the sleep mode and determines whether to jump out of the sleep mode and restart the eye tracking.
5. Mouse function module: it receives the view point of user from simulation engine and transfers the coordinate to the fixation function within user action detection module, which allows the system to perform directly at the view point after the function is selected. If a second view point is required to finish the action, the view coordinate is transferred to the simulation engine to execute the event. Besides, the module provides various virtual mouse functions for users to operate computers with eye movement. There are totally six mouse functions in this module, namely, left-click (ILEFT), continuous left-click (LEFT), double left-click (DOUBLE), right-click (RIGHT), drag (IDRAG), and scroll (SCROLL) (Figure 2).
6. Keyboard function module (KEYBOARD): provides virtual keyboard functions for users to enter text by eye movements.

### 3.2. Workflow

**Figure 3:**
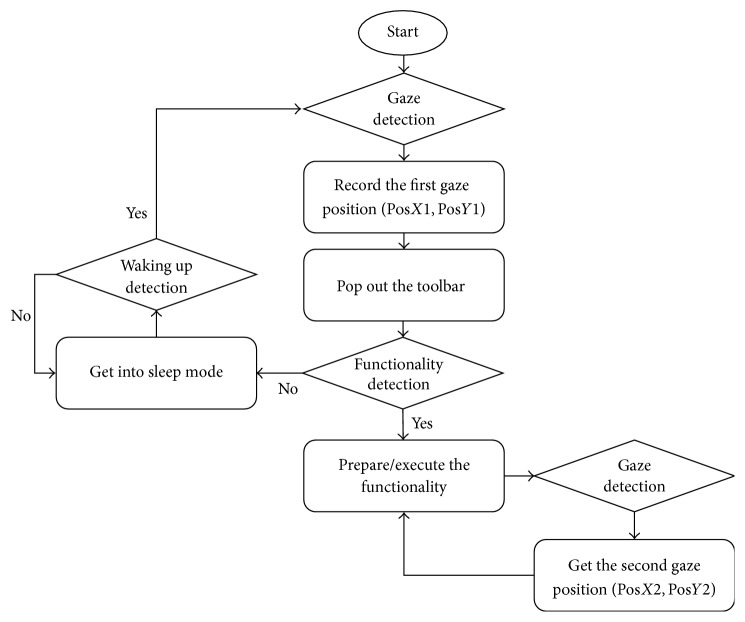
The workflow of the proposed system.

In our system, the mouse cursor is applied to direct the eye movement trajectory. Therefore, the mouse coordinates are counted and recorded every 50 ms for tracking eye movement. An effective gaze (a click) will happen if the distance of the two adjacent counts does not exceed 50 pixels for continuous 50 counts. In this way users can point and click. It is considered that users want to take actions at the coordinate of the 50th mouse records. Therefore, the current mouse coordinates were stored and then transferred in our system. The workflow of the proposed system is presented in Figure 3. First, function detects user gaze. If an effective gaze is detected, the 50th mouse coordinate is stored as the operation point. Meanwhile, the toolbar pops out at the point waiting for the user to further select the mouse function. Secondly, the gaze detection model detects whether the user has gazed on the specific tool. If the tool is selected from the tool bar, the color of the box for this tool will change from gray to white. Additionally, the system will call out the function module and perform the corresponding operation. If a second effective gaze is required to complete the tool function, the system will wait to record the second gaze position. Once gaze detection module receives the second effective eye-gaze coordinate, the coordinate information is stored and transferred to the function module. If the system does not detect user's gaze selection of the tool after toolbar has popped out for more than 2.5 s, it will send the message to the main interface module to close and hide the toolbar. Meanwhile, the system gets into sleep mode, in which the tracking for mouse cursor is stopped and waits to be waked up by users. When the mouse coordinate is moved at a range out of 100 pixels from the stopped coordinate, the timer restarts and the system is waken up to tacking eye movements.

### 3.3. Capabilities and the Usage Flows

This section contains a description of capabilities for the proposed functions. When using the proposed virtual mouse, we suggest that users follow the subsequently described processing steps. First, connect eye tribe device and check user's eye condition with a calibration. Then open the mouse cursor control function in the eye tribe device. Finally, start our system and begin to gaze at the target position to do actions. The usage flow of our system functions can be simply divided into three types: single gaze type, double gaze type, and switch mode type.

For single gaze type, including left/right-click, double left-click, and keyboard function, user is required to make only one effective gaze on the target position to complete the function. We suggest two steps to this type: (1) gaze on the position you want to do the action until the toolbar pops out on the screen; (2) gaze on the function you want to select on the toolbar until the color of the function box changes from gray to white, which refers to the fact that the function event is triggered. For double gaze type, including drag function, two effective gazes on different positions are needed for users to finish the function. A third step is required: (3) gaze on the second target position on the screen. After the third step, the function event is executed. The switch mode type includes functions of continues left-click and scroll. After presenting the toolbar, the first gaze on the function box will switch on the function and the second gaze on the box will switch off the function. The details of capabilities and the usage flows of the proposed functions are illustrated as follows:

1. ILFTE: this button is designed to implement the functionality of single left-click.
2. LEFT: this button refers to continuous left-click. The system provides a switch mode (on/off) of the button for users to simplify the operation of each left-click when they want to click multiple links in succession. After toolbar appears, the first gaze on the button means switching on the continuous left-click function and the second gaze on the button means switching off the function. When the functionality is open, only one effective gaze on the response position is needed for users to complete the left-click operation, without gazing at LEFT button on the toolbar repeatedly.
3. RIGHT: this button is designed to implement the functionality of single right-click.
4. DOUBLE: this button refers to a double left-click. It is designed for users to open a folder or execute a program quickly and efficiently.
5. IDRAG: this button is designed for users to drag and drop files or select text. The system needs to get two effective gazes to complete this action. For example, to drag and drop files, users are requested to plan steps as follows: gazing on the file icon on the screen, selecting IDRAG button in the toolbar, and gazing on target moving position on the screen.
6. SCROLL: this is designed for users to scroll pages. The switch mode is provided to turn on/off this function. During this process, the toolbar will automatically reset locations to the edge of the screen, a reading mode, which will not interfere with the reading experience of user. When user gazes on the upper part of the one-fourth of the page area, this page will scroll down. In reverse, this page will scroll up when user gazes on the lower part of the one-fourths of the page area. The page will stop scrolling if user gazes on the middle part of the two-fourths of the page area. When finishing using scroll function, user will just gaze back on the button to turn off this function.
7. KEYBOARD: this function provides a virtual keyboard interface for users to use the eye movement to enter text. The virtual keyboard is done using the source code published by MastaLomaster [16]. It is a virtual keyboard containing all the functions of the keys from the “QWERTY” keyboard. Once user gazes at key's area; a progress bar will appear in the top of the key box and countdown is started. After the countdown the key's function will automatically be executed. If user wants to close the virtual keyboard, he/she will gaze on the toolbar to turn off the function.

## 4. Experimental Results and Discussions

In experimental phase, we performed two experiments with different levels of complexities in order to evaluate the usability and reliability of the system. In the first experiment, through a searching task, we tested the system for a series of operations, like search, copy, paste, and so forth. Technology Acceptance Model (TAM), introduced by Davis [23], was used to assess user's system acceptance and understand the reason why people accepted or rejected the proposed system. In the second experiment, we tested user's acceptance of the virtual mouse on a simple and easy browsing task. Both the novel system and the previous system were tested and questioned by TAM measurements in this procedure. In addition, the statistical method of analysis was used to compare users' acceptance between the proposed virtual mouse and other virtual mouse systems. Finally, the System Usability Scale (SUS) was administered to evaluate the satisfaction of user usability of the proposed virtual mouse. Totally, 46 subjects took part in our experiments. All of them had normal or corrected-to-normal vision, and they had no prior experience with eye tracking and needed a minimal training for system using.

In order to verify the usability of our system, we use TAM questionnaire and SUS questionnaire to collect user's perception of the virtual mouse system. TAM uses theory of reasoned action as a theoretical basis for specifying the causal linkages between two key beliefs: perceived usefulness and perceived ease of use and users' attitudes, intentions, and actual computer adoption behavior [23]. In our TAM measurements, our scale items for perceived usefulness (PU), perceived ease of use (EOU), attitude towards using (ATU), and behavioral intention to use (BI) with regard to the system were adopted from Davis et al. [24] and Venkatesh and Davis [25]. We defined PU as the degree to which a person believed that using the system enhanced his or her performance. EOS was defined as the degree to which a person believed that using the system was free of effort. In addition, ATU was defined as user preferences when using the system. BI was the extent to which user would like to use the system in the future. In addition, a user would be likely to use the system because the interacting process yields fun and enjoyment [26]. Thus perceived interest (PI) was also regarded as a construct to verify the usability of system. PI was measured by items adapted from Venkatesh et al.'s work [27]. Responses to each item were measured on a 5-point Likert scale, ranging from “disagree strongly” (1) to “agree strongly” (5). At the end of the session, participants were asked to complete the SUS questionnaire [28], a standard usability measurement tool typically used to measure a user's attitudes and system preferences. It is technology independent and widely used by the worldwide developers of hardware, software, website, and so forth. The questionnaire comprised 10 questions, and each item was scored from 1 (strongly disagree) to 5 (strongly agree). The result of the questionnaire is a value between 1 and 100. Participants' responses to the SUS questionnaire proved to be a valuable evaluation tool, being robust and reliable.

In preparation for the experiment, a desktop computer with 22-inch flat screen at a 1920*∗*1080 resolution LCD was used. The virtual mouse program was created. Mouse cursor control functions were realized with an Eye Tribe Tracker device, which is cheap but with good accuracy. A document of the experimental tasks was created to guide the participants in the experiment.

### 4.1. Searching Experiment

In this part, we measure user acceptance of the proposed virtual mouse by TAM. Twenty-six participants, consisting of 11 males and 15 females and ranging from 17 to 40 years old (M = 24.77, SD = 5.78), were involved in this task. At the beginning of the experiment, participants were given an introduction to the virtual mouse. Then they were allowed to spend about 5 minutes to practice the virtual mouse by themselves. After the practices, participants were instructed to assume a comfortable position and not move too much, to maximize the eye movement tracking's accuracy. Then, a calibration with the eye tracker followed. Participants then were ready to start solving the tasks with the proposed system. During the task, volunteers were only allowed to use the proposed application to carry out the experimental process without mouse and keyboard devices. Then they were asked to fill the questionnaire of TAM for the proposed virtual mouse system, by which we can predict and explain user acceptance and rejection of the proposed system. The processes of the searching task are shown as follows:

- Please enter the home page of “IEEE Explore” by keying in “IEEE” in Google and clicking search button.
- Please search the article “An Advertisement Video Analysis System based on Eye- Tracking” by copying the title from the task document to the search field of IEEE Explore.
- Enter the article by clicking the article title in the search results.
- Find the “Date of Conference” for the article on the page, and paste the date of publication below.

The process allows the participants to use various eye control functions, such as left-click, right-click, scrolling, antiwhite, and virtual keyboard function. The TAM results of using the proposed system in searching task are shown in Table 1. The mean values of each construct are between 2.70 and 3.59 and the standard deviation values of each construct are between 0.92 and 1.23. Among these constructs, the mean value of PU (2.70) is relatively low, indicating that participants generally believe that the use of the proposed system may not be helpful in complicated searching task. The first two high scores of the mean value were credited to PI in the first place and ATU in the second, respectively, 3.59 and 3.27, implying that generally participants like the interface and operation of the proposed tool and have fun in using this tool. In addition, the mean values of EOU (3.05) and BI (3.05) are more than three, indicating that participants generally agree with the fact that the proposed system is easy to use and they are willing to use it in the future.

### 4.2. Browsing Experiment

In this section, we made comparisons of performances between the proposed system and the previous system [16] from both subjective (TAM questionnaire) and objective evidences (operation time). In addition, we also asked users to fill out SUS questionnaire on our system to evaluate the user satisfaction of the system in browsing task. Twenty new participants, consisting of 9 males and 11 females, ranging from 19 to 33 years old (M = 25.60, SD = 4.91), were involved in this procedure. Each participant had used both the systems, with a random using order. Similar to searching experiment procedure, participants were instructed in the system, had a practice with the system, and obtained a calibration with the eye tracker before task beginning. Participants were first instructed to complete using one of the random assigned systems and fill in the questionnaire to the system. Then they were allowed to use the other system and finish the questionnaires. The processes of the browsing task are shown as follows:

- Please select the webpage (such as social platform, online text) you want to browse.
- Please use the virtual mouse function to browse your chosen page for fifteen minutes.

This task allows the participants to frequently use the basic eye control functions, such as left-click and scrolling.

#### 4.2.1. Performances

The TAM results for the proposed system in browsing task are shown in Table 2. In browsing task, the means of each construct are more than those in searching task, implying that participants consider that the tool is more suitable for browsing than searching. Similar to searching task, in the browsing task the first two high scores were also credited to PI (4.26) and ATU (4.28), which implies that participants generally like the system and have fun using it in both tasks. As for the remaining dimensions, PU, EOU, and BI, they all have the mean scores higher than three, respectively, 3.45, 3.69, and 4.20, implying positive perceptions towards the proposed system.

#### 4.2.2. Comparison Results

*Subjective Comparison Results. *To assess the statistical significance of differences between the virtual mouse systems, the Wilcoxon signed-rank test was used. This nonparametric test was used in place of a more traditional analysis of variance approach because the sample size is small. Table 2 shows the results of the Wilcoxon signed-rank tests. As shown in Table 2, the proposed system experiences significantly higher value at the PU. The mean value of the proposed system is 3.45 as compared to 2.95 of the compared system. It implies that the proposed system is considered to be more useful than the compared system [16] during simple operating in browsing system. Statistically significant differences were also found for items, such as PI, ATU, and BI. The proposed system experienced an average of 4.26, 4.28, and 4.20, as compared to 4.11, 3.83, and 3.88 for the compared system, respectively. These indicate that in general participant perceived the proposed system as more advantages to use in browsing task. Though no significant difference was found on EOU between the proposed system and compared system, participants generally considered the proposed system easier to use than the compared system.

*Objective Comparison Results. *To obtain objective evaluation data, we recorded the operation times of scrolling action from 5 participants to compare between the proposed system and the compared system [16]. The time it takes for the user to successfully open the scrolling function is recorded.  Table 3 shows the comparison results of the time taken to start up the SCROLL function with both eye control systems. The average time taken to open the SCROLL function of the proposed system is 3.4 seconds, as compared to 7.8 of the compared system. It implies that the proposed system is more time saving than the other systems in browsing.

#### 4.2.3. System Usability Scale

At the end of this session, participants were asked to complete SUS questionnaire.  Table 4 presents the results of the SUS questionnaires. The system achieves a SUS score of 73.75, which is between “Good” and “Excellent” according to Bangor's research [Bangor, 2009], showing that users generally believe that the system has an excellent performance in the context of browsing data.

The standard deviation for each of the items is close to or less than one, indicating that the participants' opinions are similar. As shown in Table 4, all the means of the odd items are higher than 2.5, which means that most participants are willing to give high and positive score. Among these odd items, item 5, item 7, and item 9 obtained scores more than three, which implies that most participants believe that the functions of the system are well integrated and easy to use. Among the even items, all the means of the items are lower than 2.5, which is a good situation for our system. This reveals that this system is not difficult to use and the participants are able to use this system without much technical help. Overall, the feedback from the participations shows that they are satisfied with the proposed virtual mouse system.

## 5. Conclusions

In order to make user interact with computer naturally and conveniently by only using their eye, we provide an eye tracking based control system. The system combines both the mouse functions and keyboard functions, so that users can use our system to achieve almost all of the inputs to the computer without traditional input equipment. The system not only enables the disabled users to operate the computer the same as the normal users do but also provides normal users with a novel choice to operate computer. According to our TAM questionnaire analysis, the participants considered our eye movement system to be easy to learn. Meanwhile, participants show their interest in using the proposed eye control system to search and browse information. They are looking forward to see more of our research results on the use of eye tracking technique to interact with the computer.

In browsing experiment, the proposed system improves the browsing efficiency and experience, and with the system user can interact with multimedia with little effort. Comparing with the previous system, the participants generally prefer to use our virtual mouse system in system interface design and operation experience. The proposed system is shown to be more effective and time saving than the compared system according to the objective evidence. In the SUS questionnaire section, the participants consider that the system is of high integration functions in a simple way. In addition, the operations of the system are easy to learn and use in the integration with Internet and multimedia. Currently, this system is applied for the general operating behavior to interact with computer by simulating mouse and keyboard. In future, we will try to add new operation functions for more usage situations for users to communicate with media and adjust our system on new platform, such as tablet or phone. We will also develop series operation modules in order to achieve a complete operating experience for users from turning on to turning off the computer.

## Figures and Tables

**Table 1:** TAM results of the searching task.

Constructs (*n* = 26)	Proposed system	
	Mean	SD
PU	2.70	1.21
EOU	3.05	1.03
PI	3.59	.92
ATU	3.27	1.11
BI	3.05	1.23

Total	3.13	1.10

**Table 2:** TAM results of the browsing task.

Constructs (*n* = 19)	Proposed system		Compared system [16]		*p* value
	Mean	SD	Mean	SD	
PU	3.45	1.04	2.95	.98	0.015^*∗*^
EOU	3.69	1.06	3.53	1.08	0.365
PI	4.26	.70	4.11	.78	0.047^*∗*^
ATU	4.28	.77	3.83	.83	0.010^*∗*^
BI	4.20	.81	3.88	.83	0.044^*∗*^

Total	3.98	.88	3.66	.90	0.008^*∗∗*^

^*∗*^ *p* < 0.05. ^*∗∗*^*p* < 0.01.

**Table 3:** Comparison results of times taken to start up the SCROLL function.

Participant ID	Proposed system (second)	Compared system [16] (second)
1	3	5
2	6	16
3	2	5
4	3	5
5	3	8

Average time	3.4	7.8

**Table 4:** SUS results of the browsing task.

System Usability Scale		Mean	SD
1	I think that I would like to use this system frequently.	2.80	1.15
2	I found the system unnecessarily complex.	1.90	0.64
3	I thought the system was easy to use.	2.95	0.94
4	I think that I would need the support of a technical person to be able to use this system.	2.25	1.02
5	I found the various functions in this system were well integrated.	3.15	0.93
6	I thought there was too much inconsistency in this system.	2.10	1.02
7	I would imagine that most people would learn to use this system very quickly.	3.10	0.64
8	I found the system very cumbersome to use.	2.10	0.97
9	I felt very confident using the system.	3.00	0.86
10	I needed to learn a lot of things before I could get going with this system.	2.15	0.93

SUS score		73.75	
